# On the existence of network Macroscopic Safety Diagrams: Theory, simulation and empirical evidence

**DOI:** 10.1371/journal.pone.0200541

**Published:** 2018-08-07

**Authors:** Raed Alsalhi, Vinayak V. Dixit, Vikash V. Gayah

**Affiliations:** 1 School of Civil and Environmental Engineering, University of New South Wales, Sydney, Australia; 2 Department of Civil and Environmental Engineering, The Pennsylvania State University, University Park, Pennsylvania, United States of America; Beihang University, CHINA

## Abstract

Recent studies have proposed using well-defined relationships between network productivity and accumulation—otherwise known as Network or Macroscopic Fundamental Diagrams (network MFDs)—to model the dynamics of large-scale urban traffic networks. Network MFDs have been used to develop a variety of network-wide traffic control policies to improve a network’s operational efficiency. However, the relationship between a network’s MFD and its safety performance has not been well explored. This study proposes the existence of a Macroscopic Safety Diagram (MSD) that relates safety performance (e.g., likelihood of a crash occurring or number of vehicle conflicts observed) with the current network state (i.e., average density) in an urban traffic network. We theoretically posit a relationship between a network’s MSD and its MFD based on the average maneuver envelop of vehicles traveling within the network. Based on this model, we show that the density associated with maximum crash propensity is always expected to be larger than the density associated with maximum network productivity. This finding suggests that congested states are not only inefficient, but they might also be associated with more crashes, which can be both more unsafe and lead to decreased network reliability. These theoretical results are validated using surrogate safety assessment metrics in microsimulation and limited field empirical data from a small arterial network in Riyadh, Kingdom of Saudi Arabia. The existence of such MSDs can be used to develop more comprehensive network-wide control policies that can ensure both safe, efficient and reliable network operations.

## 1. Introduction and motivation

There has been decades of work focused on aggregate models to describe urban traffic network performance at a regional level (e.g., across small neighborhoods or networks). The earliest efforts were limited due to failure to describe the full range of congested and uncongested traffic states [1–3] or inability to model regional congestion dynamics [4–8]. This topic has recently regained renewed attention due to empirical findings that suggest that well-defined relationships exist between network production (i.e., average flow or trip completion rate) and vehicle accumulation (i.e., average density or number of vehicles currently circulating within the network) [9] and the proposal of a theoretical model that suggests such relationships could be used to describe how network accumulation evolves over time [10]. These relationships between network flow and density have come to be known as network Macroscopic Fundamental Diagrams (network MFDs).

Researchers have used these network MFDs to devise a variety of regional traffic control strategies, such as perimeter flow control (otherwise known as metering or gating) [10–18], area-wide congestion pricing [19–22], space allocation [23, 24], street network design [25–28], vehicle routing [29, 30] and large-scale network evacuation [31, 32]. However, these proposed control measures can only be used to improve a network’s operational efficiency since network MFDs only describe operational performance. The existence of relationships that predict other measures of performance at the network-wide level can lead to more holistic traffic control strategies.

The existing literature suggests that relationships might exist that could relate safety performance with operational measures. Multiple studies have used surrogate measures of safety to relate safety performance (measured by crash risk or propensity) and real-time operational measures at specific locations. Microscopic surrogate safety measures identify potential conflicts that arise between individual vehicles based on their actual trajectories [33]. Examples include time-to-collision, post-encroachment time, and maximum deceleration rate, among others [34]. Macroscopic surrogate methods use high temporal resolution (e.g., around 30-sec) real-time traffic speeds, volumes and occupancy from loop detectors to infer crash likelihood using statistical methods [35–39]. Note that the reliance of macroscopic surrogate models on high resolution temporal data have limited its application to freeways.

At the other end of the spectrum, researchers have long used observational statistical studies to predict safety performance at individual facilities as a function of traffic data aggregated over long time periods. Researchers use daily traffic volumes to normalize crash frequencies to compare crash rates across different locations [40, 41].The most prevalent research in traffic safety identifies statistical models—known as Safety Performance Functions (SPFs)—to predict crash frequencies based on daily traffic volumes, in addition to other roadway features [42] [43–45]. Perhaps because of this long temporal aggregation in volume data, SPFs generally all suggest a positive relationship between crash frequencies and traffic volumes. This seems reasonable over long time periods since more vehicles traversing a single location implies more opportunities for crashes to occur, but might not hold for volumes measured in real-time. Similar positive correlations have been observed between crash frequencies at individual locations and surrogates for traffic density measured over longer time periods, specifically the average v/c ratio [46–48]. Note, however, there has been some disagreement in the relationship between crash frequency and average speed at individual locations, with some finding a positive relationship [49–52] and others not [53, 54].

Instead of relating safety performance at a specific location and with traffic measures aggregated temporally, another approach seeks to relate safety performance and traffic measures aggregated spatially (i.e., across multiple locations, such as an entire network). [55] and the following theoretical framework [56] are perhaps the only studies to date that have explored the relationship between safety and operational performance in this way. However, these studies use the two-fluid model to describe traffic performance across a network, and this model is not able to capture urban traffic network dynamics.

In light of this, the goal of this paper is to explore the existence of Macroscopic Safety Diagrams (MSDs) of urban networks that relate safety performance and operational performance (e.g., current traffic state) in urban traffic networks in a way that is consistent with the MFD-framework that can be used to also describe traffic network dynamics. A simple theoretical model is proposed to relate the risk of multi-vehicle rear-end collisions and operational traffic state considering the average space a vehicle has to maneuver in the time-space plane. This model is then used to unveil the existence of network MSDs and identify some of their pertinent features. These theoretical findings are then confirmed using both simulation and limited empirical data collected for this study, as well as in the existing research literature. The results suggest that network MSDs do exist and provide even more justification for the implementation of the types of network-wide traffic control that have been developed using network MFDs.

The remainder of this paper is organized as follows. Section 2 describes the proposed theoretical model and discusses the expected features of network MSDs. Section 3 and Section 4 provides simulation and empirical validation for the existence of network MSDs, respectively. Finally, Section 5 offers some discussion and concluding remarks.

## 2. Theory of a Macroscopic Safety Diagram (MSD)

We propose here a simple theoretical model to explore the potential relationship between safety performance (i.e., propensity of a multi-vehicle rear-end collision) and operational performance (i.e., current traffic state) in dynamic urban networks. The validity of this model will be tested using simulated and empirical data in subsequent sections.

This basic safety model posits that each vehicle has a “maneuver envelope” within which it can safely move without colliding into another vehicle. This maneuver envelop can also be thought of as the area of the time-space plane that is associated with that individual vehicle. The spatial component of the maneuver envelope is proportional to a vehicle’s spacing (s), while the temporal component is proportional to the vehicle’s headway (h). One would expect that larger maneuver envelopes would result in a lower collision risk than smaller safety envelopes, since the large maneuver envelopes imply vehicles have greater freedom of movement without a collision spatially, temporarily or both. Thus, the risk of a vehicle being involved in a crash can be expressed as being inversely proportional to spacing and headway as follows:

$$Collision\_Risk_{i}\;\sim {\left(\frac{1}{s}\right)}^{\alpha }\times {\left(\frac{1}{h}\right)}^{\beta },$$ (1)

where α and β are coefficients that reflect the relative importance of spacing and headway on the collision risk, which may not necessarily be known a priori.

In urban traffic networks, there is a distribution of headways and spacings for individual vehicles at any moment. For example, vehicles stopped at signalized intersections would have small spacings but large headways since they are not moving. Vehicles discharging from the intersection would have larger spacings as they distance themselves from one another, but smaller headaways as they discharge at the saturation flow. Ideally, the collision risk in a network could be described as the sum of the risk of all individual vehicles based on their individual maneuver envelopes. However, doing so is not analytically tractable or useful for large networks. Instead, here we propose that the average collision risk of the (many) vehicles traveling within an urban network can be described as a function of the average maneuver envelope a vehicle experiences during its trip. This model is much more analytically tractable as it focuses on average vehicle behavior instead of the behavior of each individual vehicle in the network. This average maneuver envelope may be described by the average spacing and average headway experienced, which accounts for the variation in traffic performance throughout the network.

Aggregation at such a level is already done when calibrating SPFs that relate crash frequency as a power function of daily traffic volumes. These SPFs do not account for variations for individual vehicles but yet still provide reasonable predictions for crash frequencies at individual locations.

Noting that the average spacing and headway of vehicles in a network are inversely proportional to density (k) and flow (q), respectively, the network crash propensity, (C) can be mathematically expressed as:

$$C\left(k\right)=\gamma \times {\left(k\right)}^{\alpha }\times {\left(Q\left(k\right)\right)}^{\beta }$$ (2)

where Q(k) represents the relationship between flow and density on the network (i.e., the network MFD) and γ is a scalar constant. The existence of a well-defined MFD suggests that the average traffic performance (accounting for traffic variations within the network) can be described by just average traffic density. Thus, it is likely that density can capture variations in safety performance among individual vehicles traveling within the network at the aggregate level.

Eq (2) provides a direct relationship between the network crash propensity and both the overall traffic state (density) and the network MFD, which we now refer to as the network Macroscopic Safety Diagram (MSD). The validity of this relationship will be explored in Sections 3 and 4 using both empirical and simulation data. An example is now used to illustrate some of the features expected of the MSD. This example assumes the observed MFD for Yokohama, Japan (obtained using the total network length and average trip length from [9] and the NEF equation provided in [57]):

$$Q\left(k\right)\left[\text{veh}/\text{hr}\right]=\{\begin{array}{c} 4.00\times 10^{-10}\times {\text{k}}^{3}-1.51\times 10^{-5}\times {\text{k}}^{2}+1.68\times 10^{-1}\times k,\;\;0\leq k<106.8\;veh/km\\ 8.28\times 10^{-2}-2.43\times 10^{-2}\times k,\;\;k\geq 106.8\;veh/km \end{array}$$ (3)

**Fig 1:**
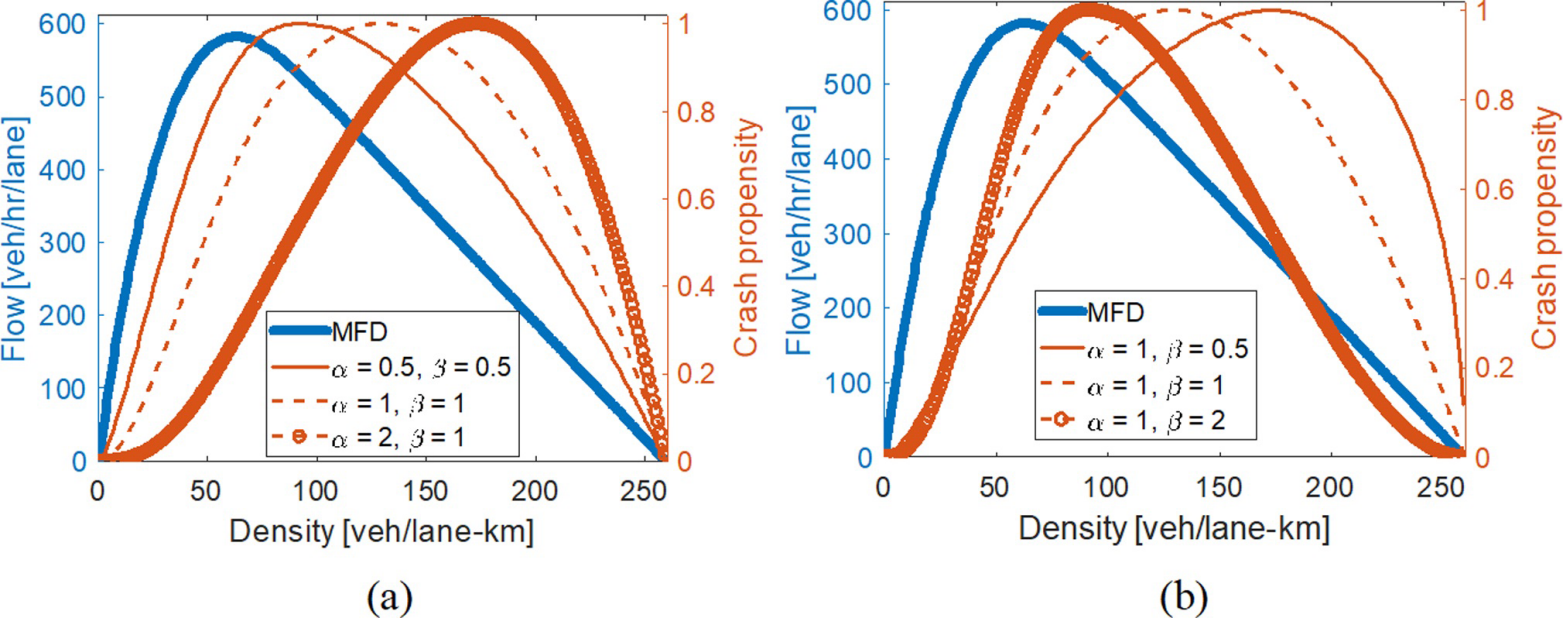
**Examples of network Macroscopic Safety Diagram using MFD of Yokohama, Japan for various values of: (a) α; and, (b) β**.

Macroscopic Safety Diagrams (MSDs) computed using (2) and (3) are provided in Fig 1 for various for various sets of α and β, where the value of γ is chosen for each curve is selected to normalize the crash propensities on the same scale between 0 and 1. These plots reveal that the network crash propensities follow a unimodal shape with zero risk at zero and jam densities. This seems reasonable as collisions would not occur when the network is empty (very left of the curve) or when the network is completely gridlocked and vehicles are not moving (very right of the curve). The unimodal shape suggests that there is a critical density that is associated with the maximum crash risk for all combinations of α and β that were considered. Furthermore, the crash propensity-density relationship is convex for low densities and then becomes a concave curve before the peak value is reached.

Interestingly, the plots in Fig 1 reveal that the critical density associated with the peak collision risk is different than the critical density associated with maximum vehicle flow. This is contrary to most prevalent crash frequency models that suggest crash frequencies increase with traffic volume [42, 44]. Specifically, we observe from Fig 1 that collision risk is greatest at a density greater than that associated with maximum vehicle flow; i.e., the peak collision risk occurs when the network is operating within a congested state. This turns out to not be a just a function of the MFD and parameters chosen, but is always true for any concave MFD and set of positive α and β values.

**Theorem 1:** For any set of positive α and β and unimodal MFD, the critical density associated with the largest crash propensity from (2) is greater than the critical density associated with maximum network flow.

**Proof:** The critical density associated with maximum network flow ($k^{*}$) is obtained as follows:

Q(k)=kv(k)

$$Q^{\prime }\left(k\right)=v\left(k\right)+kv^{\prime }\left(k\right)=0$$

$$k^{*}=-\frac{v\left(k\right)}{v^{\prime }\left(k\right)}$$ (4)

Since $v\left(k\right)$ is a positive value and $v^{'}\left(k\right)$ is a negative value for any unimodal MFD, $k^{*}$ is positive for any unimodal MFD, regardless of functional form.

The critical density associated with maximum collision risk ($k^{**}$) is obtained as follows:

$$C\left(k\right)=\gamma k^{\alpha }Q^{\beta }\left(k\right)$$

$$C\left(k\right)=\gamma k^{\alpha +\beta }v^{\beta }\left(k\right)$$

$$C^{\prime }\left(k\right)=\gamma \left(\alpha +\beta \right)k^{\alpha +\beta -1}v^{\beta }\left(k\right)+\gamma \beta k^{\alpha +\beta }v^{\beta -1}\left(k\right)v^{\prime }\left(k\right)=0$$

$$k^{**}=-\left(\frac{\alpha +\beta }{\beta }\right)\frac{v\left(k\right)}{v^{\prime }\left(k\right)}$$ (5)

Comparing (4) and (5), it follows that $k^{**}>k*$ as long as α,β>0 and the MFD is unimodal.

Theorem 1 reveals that crash propensity is highest (and thus, safety performance is worst) when the network operates within a congested state. Thus, the theoretical MSD suggests that congested traffic states should not only be avoided because they are inefficient (i.e., offer lower network flows or trip completion rates than the maximum possible) but they are also more unsafe than capacity states. Furthermore, since crashes serve as temporary bottlenecks that can significantly interrupt urban traffic network operations, these congested states that are associated with higher crash risks might also offer less reliable or predicted traffic network operations. Therefore, both network efficiency and safety performance (and perhaps even traffic network reliability) can be improved implementing control to avoid congested traffic states.

The remainder of this paper will validate the applicability of this model and the resulting trends using both simulation and empirical data.

## 3. Simulation validation

This section uses simulation data and surrogate safety measures to obtain a network’s MSD. This observed MSD is then used to validate the model proposed in the previous section that links the MSD to the network’s MFD. Section 3.1 provides a description of the simulated network and metrics that were computed to obtain the MSD, while Section 3.2 provides a summary of the results.

### 3.1. Description of simulated network and analysis metrics

**Fig 2:**
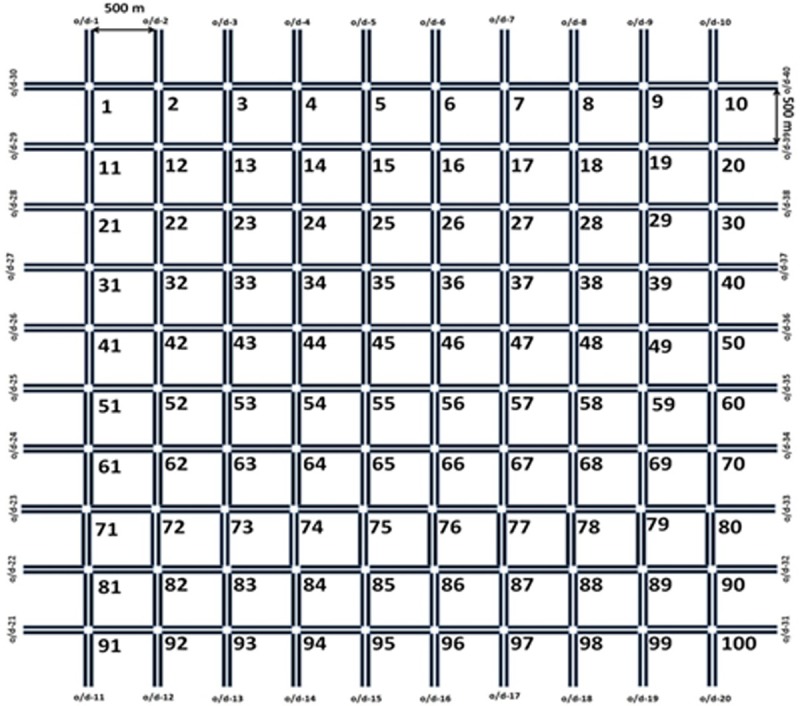
Grid network layout.

An idealized grid network was simulated in the VISSIM micro-simulation software to verify the existence of the MSD. The network consisted of a square 10 x 10 grid of identical links that were 500 meters long; see Fig 2. Each link allowed two-way travel with one travel lane in each direction. Intersections were assumed to be signalized with identical four-phase signal timing plans that provide protected left turn movements. A constant 92-second cycle length was applied and offsets between adjacent signals were set to zero. The lack of offsets are not expected to have a significant influence on the results, as a recent study revealed that favourable coordination in two-dimensional grid networks generally offer little advantage over no offsets [58].

Origins and destinations were placed at midblock locations of each internal link, as well as the entry/exit points of all links on the periphery of the network. A uniform demand pattern was adopted in which all origins and destinations exchanged the same number of expected trips, although random deviations to this occurred in each of the 15 simulation iterations that were performed. The demand gradually increased from zero over the course of the simulation to observe a range of free-flow, capacity and congested traffic conditions. Vehicles were assumed to route themselves to minimize their personal travel time and mid-trip rerouting is available for a fraction of vehicles based on observed travel times.

Individual vehicle trajectories were obtained from each of the simulation iterations. These trajectory data were used to compute the average flow, density and vehicle speed at regular 5-minute intervals for the entire network using the generalized definitions of [59]:

$$Q=\left(\frac{\sum d_{i}}{L.T}\right)$$ (6)

$$K=\left(\frac{\sum t_{i}}{L.T}\right)$$ (7)

$$V=\left(\frac{\sum d_{i}}{\sum t_{i}}\right)$$ (8)

where $\sum d_{i}$ total distance travelled by all vehicles during the aggregation interval, $\sum t_{i}$ is total time spent by all vehicles in the network, T is the length of the aggregation interval (5 minutes), and L is the total network length (110 lane-km). Eqs (6) through (8) provide the observed network MFD, which was used to compare with observed safety performance.

Safety performance in the simulation environment was quantified using the FHWA Surrogate Safety Assessment Measures (SSAM) toolbox. The SSAM toolbox uses individual vehicle trajectory information to identify vehicle conflicts using known surrogate safety measures. For this study, the surrogate safety measure considered was the minimum time-to-collision (TTC). TTC is defined as the time required for two vehicles to collide if they continue at their present speed on the same path [60]. While the TTC cannot identify crashes directly, it identifies situations in which crashes might be likely if drivers were inattentive and thus serves as a reliable surrogate for crash frequency in simulation environments [33].

Within the simulation environment, safety conflicts within each of the 5-minute aggregation intervals were quantified by identifying TTC values lower than some critical threshold. The number of conflicts is used as a surrogate for the propensity for a collision within the network. Multiple critical TTC thresholds were considered (TTC≤0.5, TTC≤1.0,TTC≤1.5 ) to examine the sensitivity of the results to the threshold value chosen. The SSAM also categorizes conflicts into one of three types (rear-end, angle, and lane-change) based on the trajectories and relative position of each of the vehicles involved in a conflict. Only conflicts identified as rear-end collisions were considered in this study.

### 3.2. Simulation results

**Fig 3:**
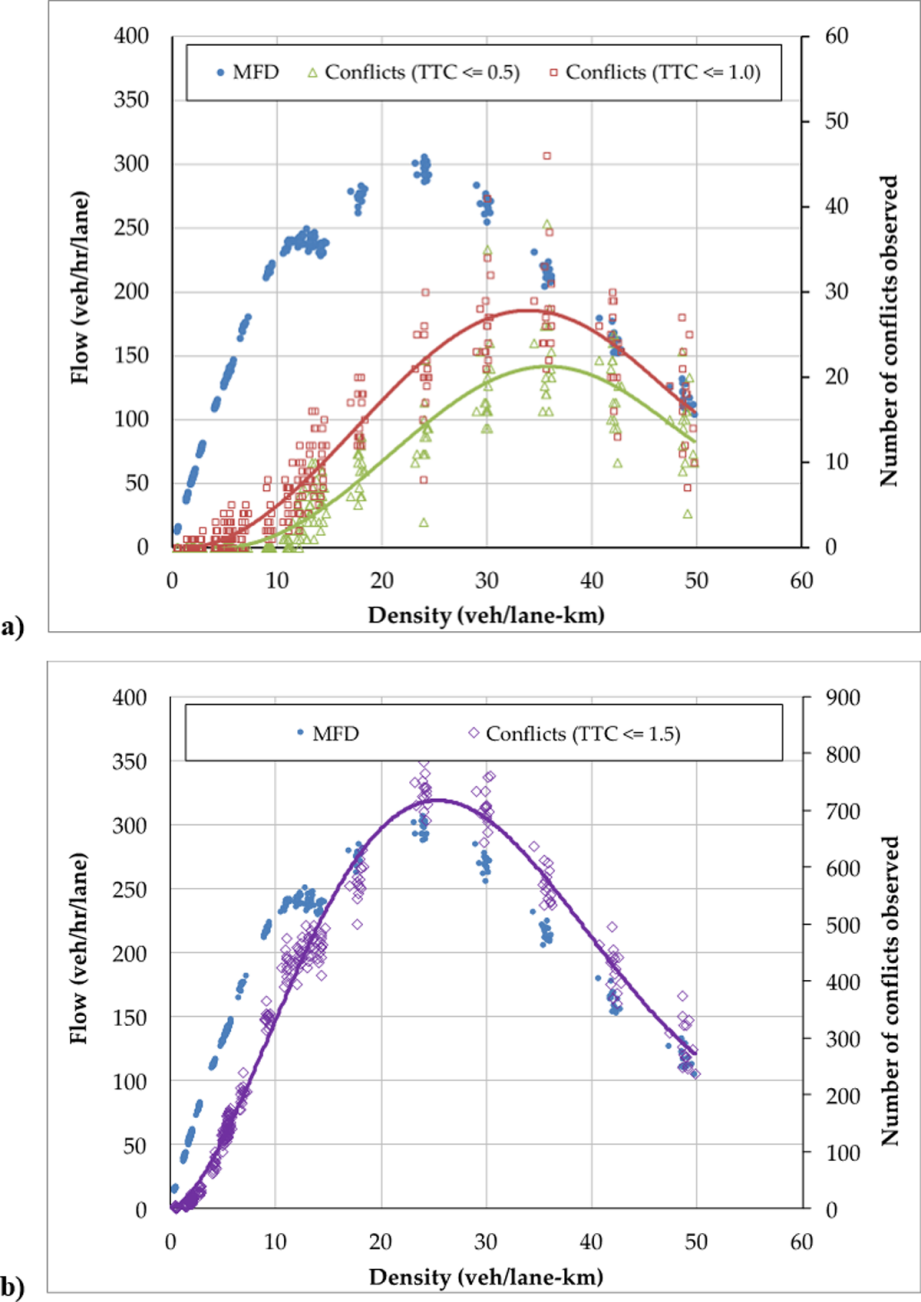
Observed flow-density (i.e., network MFD) and conflict-density relationships obtained from micro-simulation for: (a) TTC≤0.5 and TTC≤1.0; and, (b) TTC≤1.5.

The observed network MFD from the simulation is provided in Fig 3. As illustrated, the observed MFD (solid points) reveal a typical unimodal shape with no scatter in the free flow branch and relatively low scatter near capacity and congestion. The highest flow observed occurs at a critical density of about 24 veh/lane-km.

Fig 3 also provides the observed relationship between observed vehicle conflict frequency and average network density for three of the TTC thresholds considered (TTC≤0.5, TTC≤1.0 and TTC≤1.5). For both cases pictured, the individual markers represent the values observed over the 15 simulation iterations while the solid line represents a simple polynomial regression curve that best fits the observed data. As expected, the number of conflicts observed increases with the TTC threshold selected; i.e., higher thresholds provide more conflicts. The number of conflicts increases significantly when moving from TTC≤1.0 to TTC≤1.5, which reflects the sensitivity of the surrogate measure to the threshold value in this range.

What is interesting is that the relationship between observed vehicle conflict frequency and density follows a unimodal pattern in all cases. The frequency of conflicts is zero at zero density and increases with density in a convex relationship for low densities. This is reasonable as in light traffic vehicles interact less frequently and thus do not have many opportunities to collide. For moderate densities, the relationship between conflict frequency and density is concave with a well-defined peak. This also makes sense since vehicles move more slowly and with larger headways on average as the network gets congested; thus, fewer crashes would be expected when the network is very congested. Remarkably, for all TTC thresholds chosen and all simulation runs, the peak observed conflict frequency occurs at a critical density that is greater than the density that is associated with maximum network flow. This critical density associated with maximum observed conflict frequency is in the range of 27–36 veh/lane-km and decreases with the TTC threshold selected. This latter fact is reasonable, since a vehicle’s TTC is related to its headway and higher network flows are associated with smaller average headways. Additional simulations (not shown here) verify that for various network configurations and signal settings, a unimodal pattern was always observed between number of vehicle conflicts and average network density in the simulated environment and that this pattern was also independent of the TTC threshold chosen.

These observed patterns and findings are consistent with the simple theoretical model proposed in Section 2. To further explore this relationship, including the sensitivity of the safety performance to the TTC threshold selected, we now examine how well the model proposed in (2) fits the observed simulation data. Since only observed conflict data is available in the simulation environment, the crash propensity in (2) is replaced with the number of conflicts observed for each TTC threshold. Non-linear regression is used to estimate the model parameters for each of the four TTC thresholds considered. The parameter estimates, associated standard errors, and goodness-of-fit statistics ($R^{2}$ and sum of squared errors) are provided in Table 1. The number of observations included in the regression model is 360 in each case (24 observed densities in each of the 15 simulation iterations).

In all cases, the proposed theoretical model shows an extremely good fit to the observed simulation data, as reflected by the $R^{2}$ values exceeding 0.90 in all cases. As expected, the parameter estimates for α, β and γ (referred to here as $\gamma_{conflicts}$ to represent the use of vehicle conflicts as the dependent variable instead of crash propensity) are all positive. The parameter estimates for both α and β are statistically significant in all models, which suggests statistically significant relationship between operational parameters (network density and flow) and safety performance (as reflected in the observed vehicle conflicts). The range of parameter estimates observed across the thresholds reveal that the MSD relationship is sensitive to the critical threshold chosen in the simulation environment. This seems reasonable as the number of conflicts observed increases significantly with the TTC threshold chosen, especially as the TTC threshold increases beyond 1.0.

**Table 1:** Parameter estimates for theoretical model applied to simulated data for various TTC thresholds.

	TTC≤0.5		TTC≤1.0		TTC≤ 1.5	
	est.	std. error	est.	std. error	est.	std. error
α	1.987415	0.886227	1.314498	0.044663	0.402045	0.005612
β	1.545900	0.085452	1.249237	0.058487	1.349833	0.012966
$\gamma_{conflicts}$	4.09E-06	2.98E-06	2.90E-04	1.24E-04	9.56E-02	6.82E-03
$R^{2}$	0.9208		0.9417		0.9968	
Sum of sq. errors	2148.098		3056.739		141548.3	

We now apply these model results to compare the theoretical derived MSD with the observed relationship between network density and vehicle conflicts obtained from the simulation. Non-linear regression is used to fit a simply third-degree polynomial to the observed MFD, which provides the following relationship between observed flow and density:

$$Q\left(k\right)=0.0079k^{3}-0.9567k^{2}+30.253k$$ (9)

**Fig 4:**
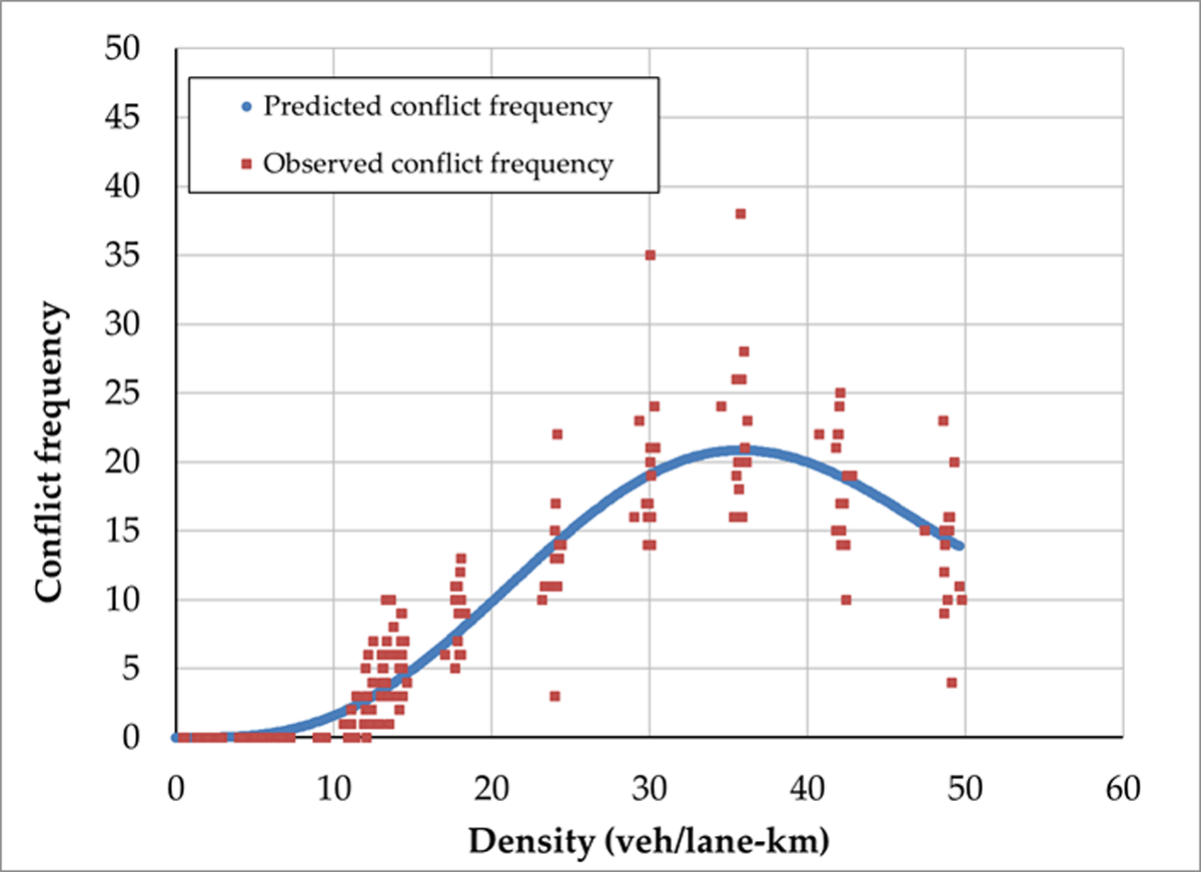
Observed and predicted conflict-density relationships (i.e., MSDs) obtained from micro-simulation.

The parameter estimates for the critical threshold TTC≤0.5 are then used to derive the theoretical MSD relationship expected. Fig 4 reveals that the predicted conflict frequency using the theoretical model matches very well with the observed frequency. Specifically, the predicted relationship between safety performance and average network density follows the same general shape and peaks at the same density (about 35 veh/lane-km) for the TTC threshold chosen. Similar results hold for all TTC thresholds considered. Overall, these simulated results suggest that the proposed theoretical model accurately describes the relationship between safety performance (measured by vehicle conflicts) and traffic density that is observed in the simulation environment.

## 4. Empirical validation

This section provides additional validation for the existence and shape of a network’s MSD using empirical data. Section 4.1 describes the empirical data that were used obtained and used as a part of this validation, and Section 4.2 provides a summary of the results.

### 4.1. Description of empirical data

**Fig 5:**
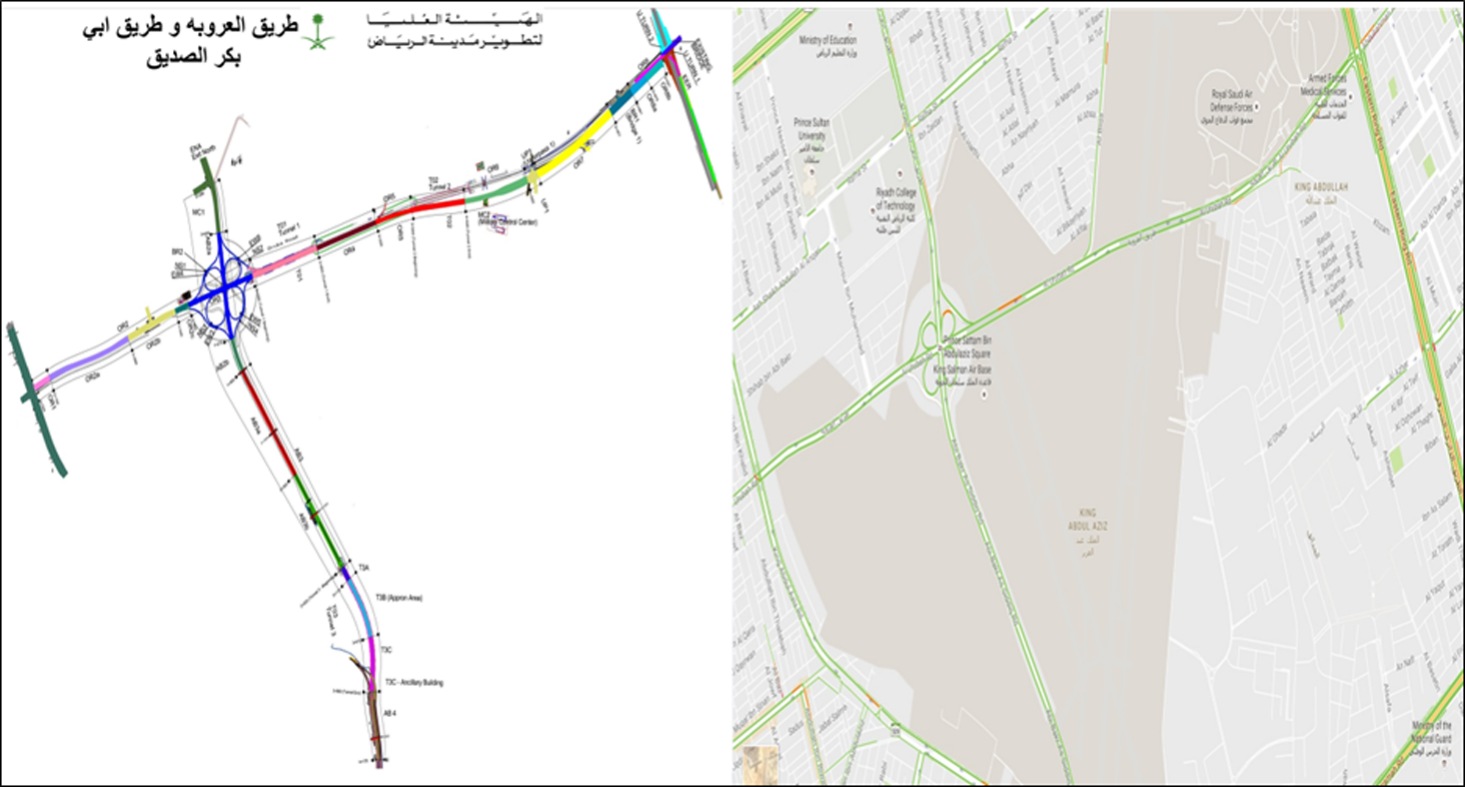
Study site in Riyadh, Kingdom of Saudi Arabia.

Traffic flow and crash data were obtained from a small arterial network in Riyadh, the capital and largest city of the Kingdom of Saudi Arabia. The arterial network consists of just two intersecting arterials—Al Urubah Road and Abu Bakr Al Siddiq—connected by a signalized intersection as shown in Fig 5. Each of the two arterials has three 3.6 meter-wide travel lanes in each direction.

A portion of this arterial network (about 5 km on Abur Bakr Al Siddiq and 6 km on Al Urubah Road) is continuously monitored using a closed-circuit television (CCTV) system containing 260 fixed camera and 34 coaxial cameras. This CCTV system is used to obtain vehicle flows and average travel speeds at each of the 260 fixed camera locations within the network at regular 15-minute intervals throughout the day. For the purposes of this study, CCTV traffic data from the year 2015 was obtained from Saudi Arabia’s Arriyadh Development Authority (ADA) for the monitored section of this arterial network. This dataset consisted of 9,110,400 unique observations, representing an observation from each of the camera locations and 15-minute periods throughout the year. Although 15-minute measurement intervals might reduce the ability to capture short-term variations in traffic conditions, smaller aggregation intervals were not possible due to technical limitations.

Safety performance data was also obtained from the ADA. This safety data consisted of all observed crashes within the monitored segment for the same time period (the year 2015). Each crash was identified from the CCTV system and a detailed record of the date, time and location of the crash (with respect to location on the roadway and camera that observed the crash) was available. These observations consisted of both serious (fatal or injury crashes) and non-serious (property damage-only) crashes. While the latter might be less critical for safety applications, they are still vital as they can disrupt operations along the arterial network. A total of 728 crashes were observed during the year 2015 analysis period.

### 4.2. Data cleaning

Traffic and crash data were first filtered to remove observations that occurred on the interchange ramps that connect the two arterials. These ramp locations have reduced speeds and did not reflect mainline traffic conditions and were thus excluded from the analysis. The data were then filtered to remove any abnormal or erroneous observations. This included observations with either positive flows and zero speeds or positive speeds and zero flows. Time periods that had erroneous data anywhere in the network were removed entirely from the dataset. Speed and flow data were then used to estimate average density at each of the monitored sections using the fundamental traffic relationship. Finally, flow, speed and density data were aggregated to obtain average values across the arterial network consistent with previous definitions in Section 3.1. After the processing stage, a total of 34,037 average flow, density and speed observations were available for the entire arterial network considered. This represented 2.86% reduction over the total possible 35,040 observations that were possible for the year 2015.

Crash data were also filtered to remove observations at the ramps connecting the arterial roadways. Crashes that were observed without any corresponding traffic data at that location or for which traffic data were missing from some portion of the network were also removed. The final dataset consisted of 605 crashes, which represented 83% of the total 728 available. The majority of the crashes that were removed occurred at the ramp locations.

### 4.3. Time-series analysis

**Fig 6:**
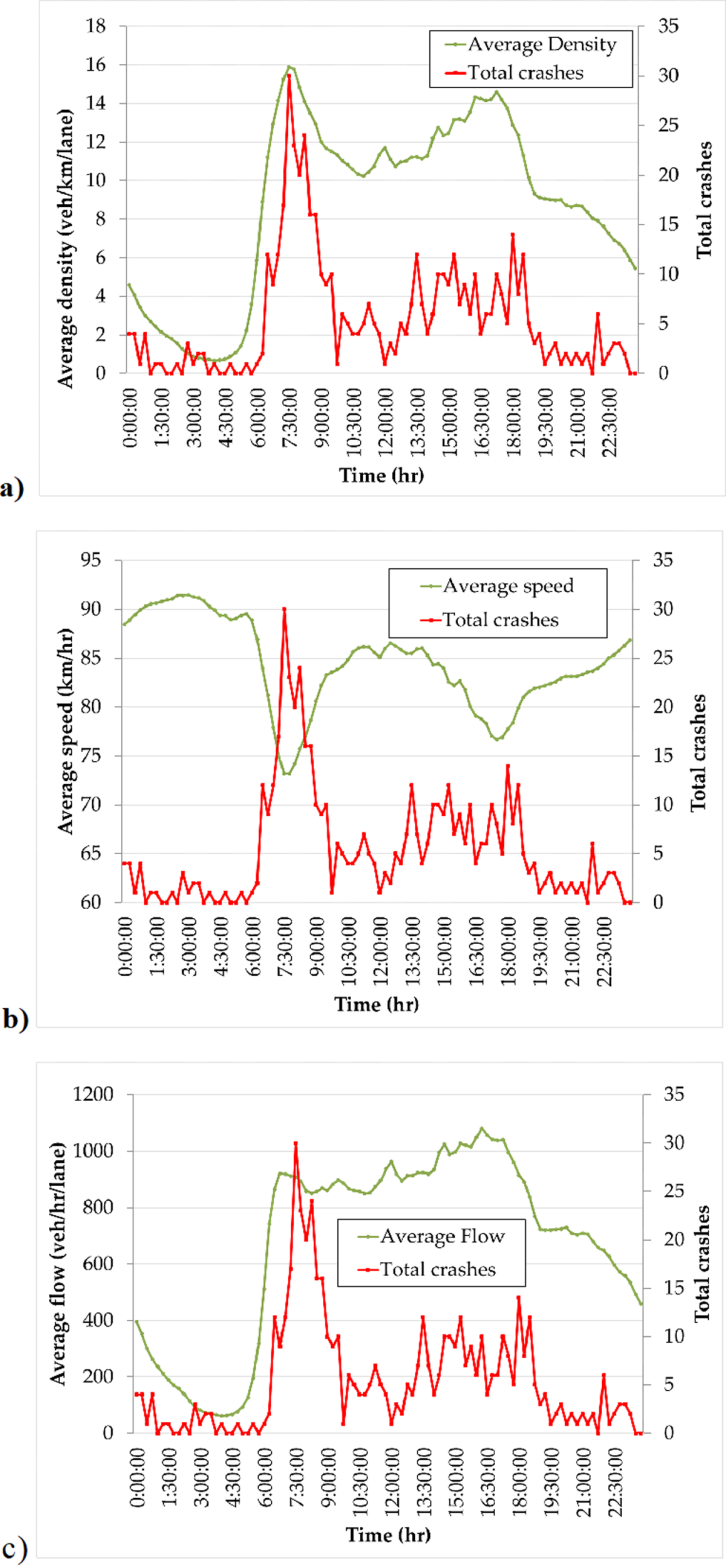
Time series plot of crash frequency vs. a) average network density; b) average network speed; and, c) average network flow.

Time series plots of average network density, speed and flow were compared with observed crash frequencies to explore if well-defined relationships exist between safety and operational performance. Fig 6 provides these relationships for the weekdays only (Sunday–Thursday in Saudi Arabia), since average densities, speeds and flows were fairly consistent during weekday periods.

As shown in Fig 6A, observed crash frequency shows a somewhat positive correlation with average network density. Few crashes occur during the early morning hours when no congestion is present. Density and observed crashes appear to spike during 6AM to 8AM time period, which coincides with the morning rush. Crash frequency subsides with the tail of the morning rush period (from about 8AM to 10 AM) and remains fairly consistent until the PM peak period. The correlation between crash frequency and density is not as strong during the PM peak period; however, this PM peak is characterized by a much less sharp increase in density over time. Thus, we see that the relationship between crash frequency and average density might indeed follow a non-linear relationship as suggested by the model proposed in Section 2.

Fig 6B reveals similar but negative correlation between average network speed and crash frequency. This seems reasonable since when a network exhibits a well-defined, concave network MFD, a one-to-one relationship exists between average network density and average network speed and speed decreases with average network density. Fig 6C shows a less strong correlation between average network flow and crash frequency during the day. Specifically, although flow and crash frequency peaks during the early PM peak period, no noticeable drop in flow is observed at the end of the AM peak period even though crash frequency drops significantly.

Pearson’s correlation coefficients were computed for each of these operational measures and observed crash frequencies. The correlation values were 0.694536, -0.75053 and 0.552162 for average network density, speed and flow, respectively. Overall, these results are consistent with Fig 6. They also our hypothesis that a network MSD exists since congestion seems to be associated with higher observed crash frequencies.

### 4.4. Observed network MFD and MSD

**Fig 7:**
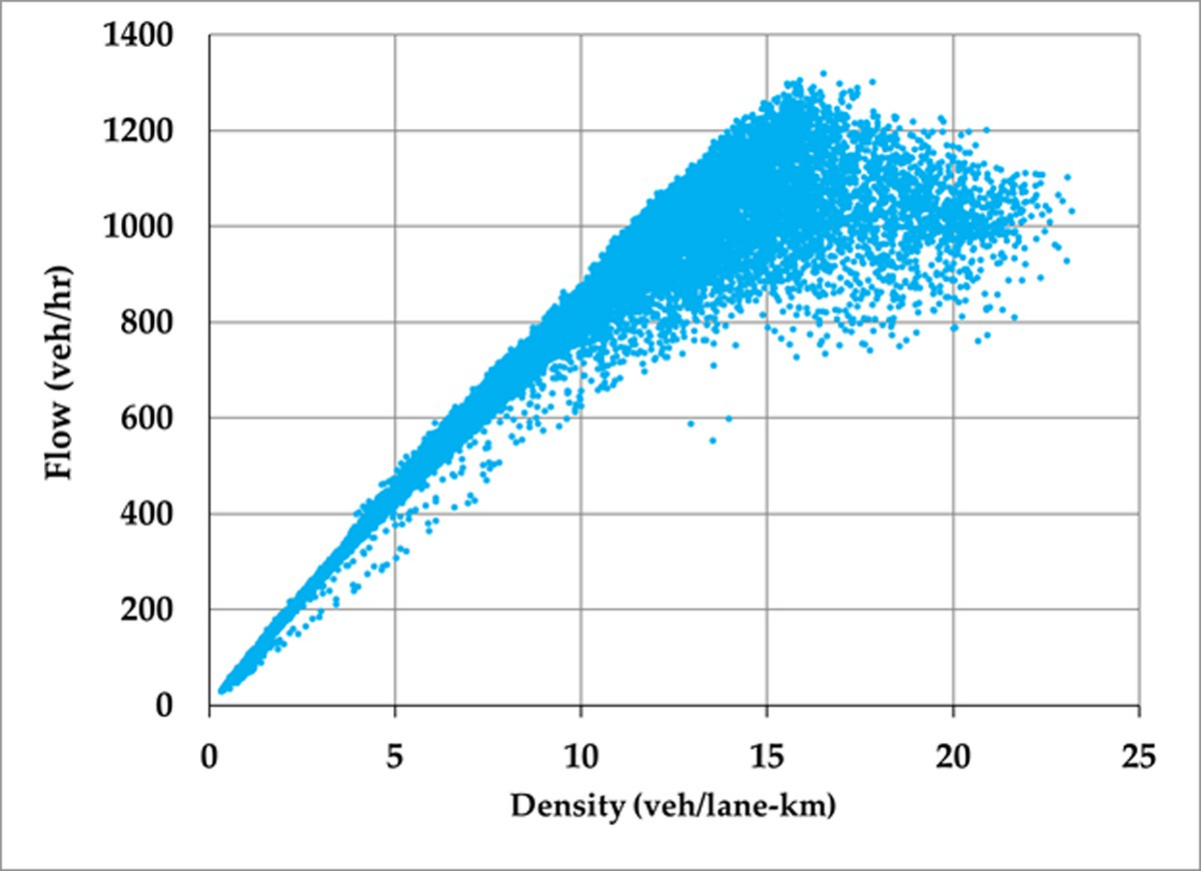
Observed network MFD for arterial network.

The observed relationship between average network flow and average network density (i.e., the observed network MFD) for the arterial network is provided in Fig 7. Large amounts of scatter exists because traffic conditions are not homogeneous across the arterial section during each 15-minute interval. Furthermore, the presence of queues on the ramps that connect the two arterial segments could influence traffic operations and contribute to this scatter. Fig 7 also reveals that a full range of traffic conditions are not observed: the observed maximum density is only about 22 veh/lane-km and thus does not include highly congested or gridlocked states. Nevertheless, the relationship shows a clear concave pattern with a well-defined peak flow observed for densities in the range of about 14 to 16 veh/lane-km. Note that this is lower than the critical density for the fundamental diagram for a single location (which is on the order of about 30 veh/lane-km) since the network has heterogeneous traffic conditions and this network-wide average includes both congested and uncongested areas in the network simultaneously.

**Fig 8:**
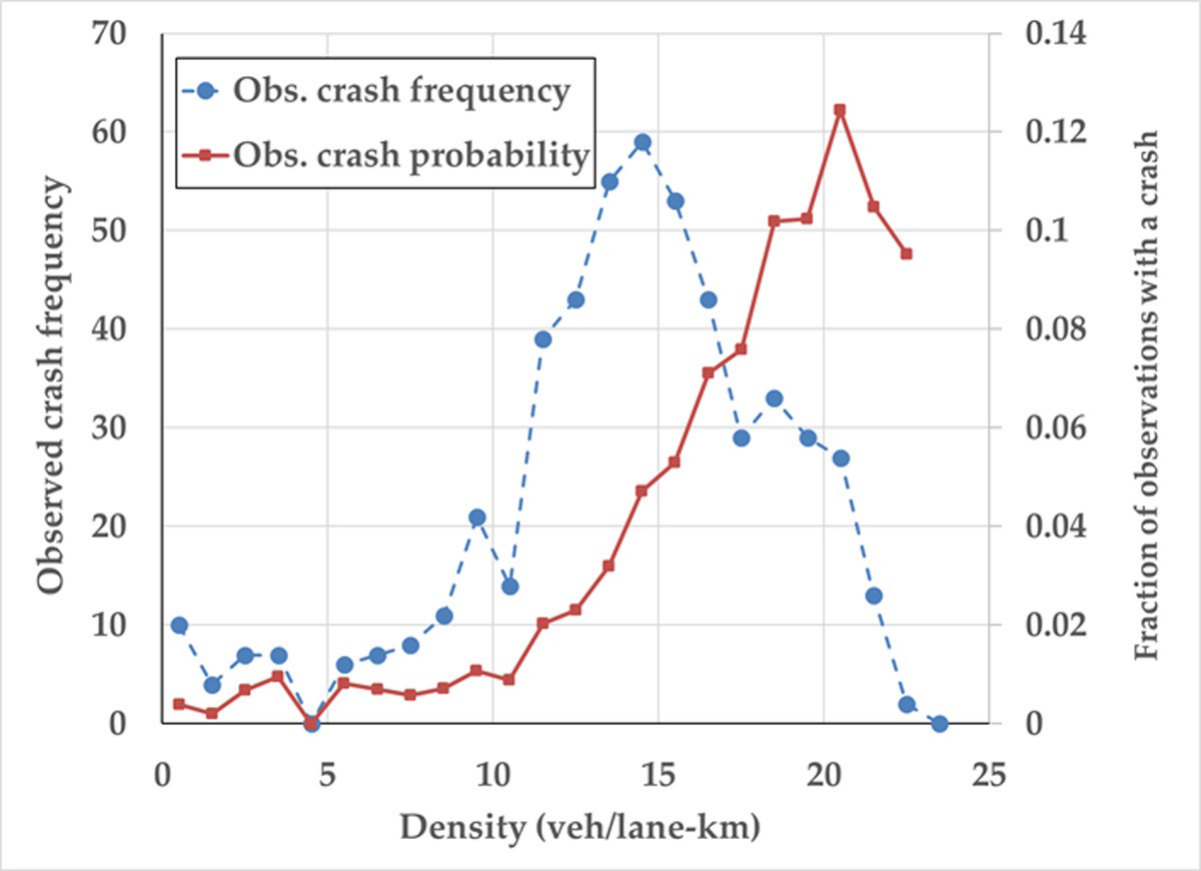
Observed network MSD. Broken line represents observed crash frequencies while solid line represents crash risk.

The observed relationship between safety performance and average network density is provided in Fig 8. Two relationships are presented: one for crash frequency (broken line) and one for crash risk or probability (solid line). In the first case, observed densities were grouped into 1 veh/lane-km bins (e.g., density of 0–1 veh/lane-km, 1–2 veh/lane-km, etc.) and the total number of crashes observed when the network operated within each of these density ranges is plotted. This observed crash frequency relationship has a more or less unimodal shape that suggests that the most crashes are observed when the network operates at a density of 14–15 veh/lane-km. This is similar to the critical density associated with the maximum network flow. However, this observed frequency relationships fails to account for the exposure of the different network densities. Specifically, lower densities are simply observed more often than higher densities since the network operates in less congested states with more frequency; see Fig 6A for an illustration. Thus, in a given time period, more crashes are likely to be observed at lower densities than higher densities simply because traffic operates at these lower densities more frequently throughout the one-year analysis period.

To better account for this exposure issue, the solid line in Fig 8 shows the proportion of 15-minute periods within each of the 1 veh/lane-km density ranges throughout the one-year analysis time period that are associated with a crash occurrence. This relationship represents the true network MSD that relates the risk of a crash with traffic network performance. This network MSD relationship reveals that the largest crash risk occurs within a density range of 20–21 veh/lane-km. Table 2 provide the parameter estimates of the theoretical model proposes in Eq (2) using the data presented in Fig 8. As shown, the parameter estimates are positive and in line with expectation. These results validate the theoretical and simulation trends that suggest safety performance peaks at a density greater than the critical density associated with maximum network flow (i.e., when the network operates within a congested state).

**Table 2:** Parameter estimates for theoretical model applied to empirical data.

	est.	std. error
α	3.029227	0.2184467
β	2.463408	0.3540539
γ	4.70E-13	1.37E-12
$R^{2}$	0.9892	
Sum of sq. errors	8.1078E-3	

### 4.5. Consistency with previous studies

**Fig 9:**
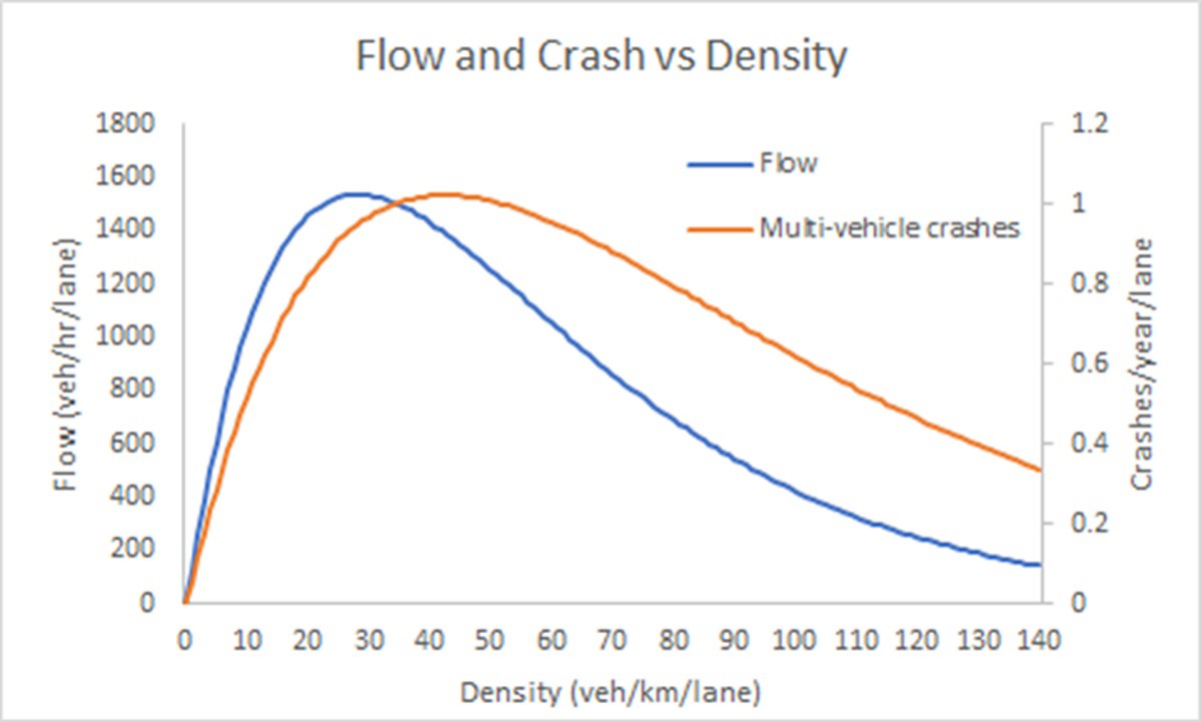
Observed flow-density and crash rate-density relationships for urban roads reproduced from [47].

An earlier study by [47] explored the relationship between average flow and density (Fig 1 in [47]) as well as the relationship between multi-vehicle crash rates and density (Eq 6 and results in Table 3 in [47]) on urban roads. Models fit to these data were estimated and the model results are reproduced in Fig 9. It can be clearly seen that the same unimodal curve predicted by our theory for multi-vehicle crashes is also observed in these data. Furthermore, as per the predictions of Theorem 1, the peak crash frequency is observed at a density that is greater than the density that maximizes the observed flow-density relationship.

**Fig 10:**
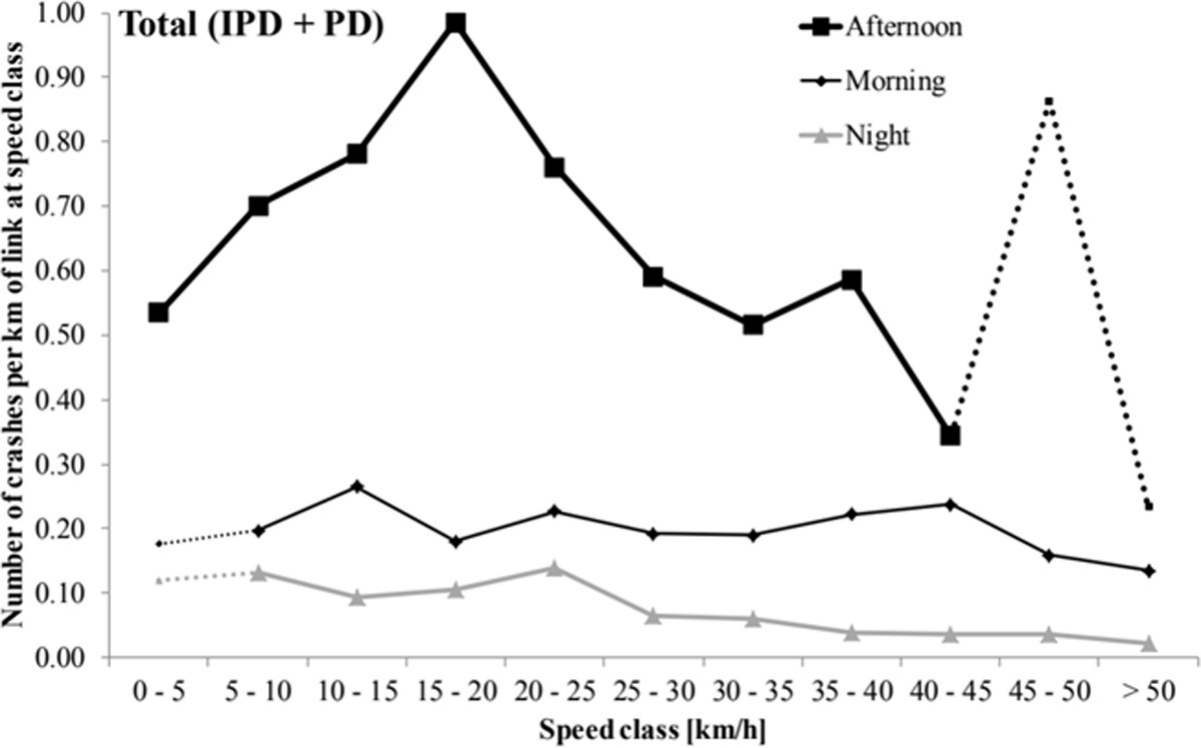
Observed crash rate-speed relationship reproduced from [61].

Another recent study explored the relationship between observed crash rates and average travel speeds [61]. The observed relationship is reproduced in Fig 10. The data reveals a unimodal relationship in which lower crash rates are observed at the lowest and highest travel speeds and the highest crash rates are observed at moderate speeds (specifically, around 15–20 km/hr). This unimodal pattern between crash rate and speed is also an outcome of the model proposed in this paper due to the fundamental relationship between flow, density and average travel speed. Overall, the results of these two empirical studies help to provide additional empirical verification of the proposed model.

## 5. Discussion and concluding remarks

This paper presents the first evidence of the existence of Macroscopic Safety Diagrams (MSDs) for urban networks, which relate the safety performance of the network (i.e., risk of a collision) to its real-time operational performance (i.e., current traffic state). A simple theoretical model based on vehicle maneuver envelopes in the time-space plane is used to illustrate that the safety performance of a network can be linked to its operational performance directly using the network Macroscopic Fundamental Diagram (MFD). Examination of this theoretical MSD model reveals that collision risk peaks (i.e., safety performance is at its worst) for network densities greater than the critical density associated with maximum network flow. This finding suggests that congested traffic states are not only inefficient, but might also be associated with decreased safety performance and disruptions in traffic flow due to more frequent crashes. This has significant implications for network-wide traffic control and management as it suggests that perimeter flow control and pricing strategies designed to keep networks from being congested might also simultaneously improve safety performance by reducing the risk of vehicle collisions and maintain more reliable network performance.

The existence of the MSD is confirmed using both micro-simulation tests of an idealized grid network and empirical data from a small arterial network in Saudi Arabia. In the micro-simulation tests, surrogate safety measures are used to identify conflicts between individual vehicles in the network, while observed crash frequencies over a one-year period are used to quantify safety performance in the empirical data. In both cases, the general shapes of the network MSDs and overall pattern related to decreased safety performance of congested traffic states are consistent with the theoretical expectations. Such shapes have also been reported in previous studies in the research literature.

Though we have validated a-priori theoretical results with empirical findings through statistical tools, we would like to posit caution on assuming MSD to be a fundamental characteristic. Statistical relationships in traffic do not imply a fundamental relationship (Ranjan et al. 2016), and more empirical evidence would be needed. It should also be noted that the empirical findings are limited by data availability. Further work is needed to examine that empirical MSD relationships also hold in these highly congested situations. This is especially important since these highly congested network states are typically characterized by inhomogeneous congestion patterns that could further influence expected safety performance. The relationship between safety performance and congestion inhomogeneity should also be further studied, especially since network MFDs have been shown to be sensitive to congestion distributions within the network. Fortunately, most networks do not operate within these highly congested states for long, as evidenced by the lack of empirical MFDs with very large average densities. Finally, the relationship between the parameters of the MSD model proposed in this paper (α, β, γ) and other network properties need to be studied. It seems reasonable that these parameters would change based on properties such as network structure and connectivity, signal settings, amount of turning manuevers (captured by origin-destination patterns) and presence (or lack) of turning lanes. Further work is needed to explore these relationships.

## Supporting information

S1 File Simulation model for the grid network. (ZIP)

S2 File Field data. (ZIP)
